# Application of T1-/T2-Weighted Ratio Mapping to Elucidate Intracortical Demyelination Process in the Alzheimer’s Disease Continuum

**DOI:** 10.3389/fnins.2019.00904

**Published:** 2019-09-10

**Authors:** Xiao Luo, Kaicheng Li, Qingze Zeng, Peiyu Huang, Yeerfan Jiaerken, Shuyue Wang, Zhujing Shen, Xiaojun Xu, Jingjing Xu, Chao Wang, Linlin Kong, Jiong Zhou, Minming Zhang

**Affiliations:** ^1^Department of Radiology, The Second Affiliated Hospital, School of Medicine, Zhejiang University, Hangzhou, China; ^2^Department of Neurology, The Second Affiliated Hospital, School of Medicine, Zhejiang University, Hangzhou, China

**Keywords:** Alzheimer’s disease continuum, inferior parietal lobule, amyloid, intracortical myelin, preclinical Alzheimer’s disease

## Abstract

**Background** The biological diagnosis criteria of the Alzheimer’s disease (AD) suggests that previous work may misclassify the cognitive impairment caused by other factors into AD. Consequently, re-assessing the imaging profile of AD continuum is needed. Considering the high vulnerability of cortical association fibers, we aimed to elucidate the cortical demyelination process in the AD continuum biologically defined.

**Methods** According to the biological diagnosis criteria, we determined the positive amyloid status (A+) as cerebrospinal fluid (CSF) amyloid_1__–__42_ < 192 pg/ml, Florbetapir Positron emission tomography (PET) composite standardized uptake value ratio (SUVR) >1.11. Also, the positive Tau status (T+) was determined as p-Tau_181_ > 23 pg/ml. Based on the cognitive characterization, we further categorized 252 subjects into 27 cognitively unimpaired with normal AD biomarkers (A−T−, controls), 49 preclinical AD (A+T+), 113 AD with mild cognitive impairment (MCI) (A+T+), and 63 AD dementia (A+T+). We estimated the intracortical myelin content used the T1- and T2-weighted (T1W/T2W) ratio mapping. To investigate the sensitivity of the ratio mapping, we also utilized well-validated AD imaging biomarkers as the reference, including gray matter volume and Fludeoxyglucose PET (FDG-PET). Based on the general linear model, we conducted the voxel-wise two-sample *T*-tests between the controls and each group in the AD continuum.

**Results** Compared to the controls, the results showed that the preclinical AD patients exhibited decreased T1W/T2W ratio value in the right inferior parietal lobule (IPL); as the disease progresses, the prodromal AD patients demonstrated lower ratio value in bilateral IPL, with hippocampus (HP) atrophy. Lastly, the AD dementia patients exhibited decreased ratio value in bilateral IPL and hippocampus; also, we observed the bilateral temporal cortices atrophy and widespread decreased metabolism in the AD dementia patients. After corrected with gray volume, the results remained mostly unchanged.

**Conclusion** Our study implied the decreased right IPL T1W/T2W ratio might represent early AD-related demyelination in disease continuum. Additionally, we demonstrated that the T1W/T2W ratio mapping is an easy-to-implement and sensitive metric to assess the intracortical myelin content in AD, particularly in the early stage.

## Introduction

Alzheimer’s disease (AD) is clinically characterized by significant memory impairment, accompany with other cognitive abilities decline. Concerning AD neuropathologies, extracellular amyloid plaques deposition appears the earliest, followed by intracellular neurofibrillary tangles and downstream neurodegeneration events (Braak et al., 2006). In the recent decade, the distinction between clinical symptoms and neuropathological changes became blur due to lack of neuropathological examination. Consequently, prior AD-related studies may misclassify the cognitive impairment caused by neuropsychiatric factors or cerebral vascular disease into AD (Kalaria, 2016). In 2018, the research framework made by the National Institute on Aging-Alzheimer’s Association (NIA-AA) formally advised that the term AD should be defined by pathological biomarkers *in vivo*, rather than clinical symptoms (Jack et al., 2018). Meanwhile, *in vivo* neuroimaging techniques that enable topological changes assessment has been widely used to visualize the stepwise downstream events in AD continuum (Li and Zhang, 2015; Peng et al., 2015; Perrotin et al., 2015; Stricker et al., 2016; Leijenaar et al., 2017; Mito et al., 2018). Similarly, considering the potential subjects misclassification, re-assessing the imaging profile of AD continuum defined by biological criteria is needed.

Myelin provides the neuronal basis of high processing speeds for high-level cognitive function (Felts et al., 1997). On the other side, myelinated projection and association fibers are vulnerable to the negative effects of AD neuropathologies (Bartzokis, 2004). Thus, myelin mapping is potentially an ideal biomarker monitoring progression of AD. Presently, most AD-related studies focused on myelin of deep white matter via diffusion models. However, because of higher sensitivity to injury factors, the intracortical myelin that sustains association fibers recently receives more attention (Li and Zhang, 2015; Stricker et al., 2016). Specifically, regions late to myelinate have fewer wraps, leading to its less protection against damage than the deep white matter tracts (Bartzokis, 2011; Landau et al., 2011). Accordingly, at the foundation of myelin model proposed by Bartzokis, the AD-related myelin breakdown follows the myelination developmental process in reverse, roughly from the association fibers to the deep projection tracts, and from the posterior to the anterior regions (Bartzokis, 2004; Bartzokis et al., 2007). Though clinical significant, mapping intracortical myelin in humans is still a challenge. Anatomically, crossing cortical association fibers impede the application of traditional diffusion-weighted imaging (DWI) (Phillips et al., 2016). By contrast, recently proposed the T1-weighted/T2-weighted (T1W/T2W) ratio mapping takes advantage of the evidence that myelin content tightly linked with both T1W and T2W intensity, but in opposite directions (Glasser and Van Essen, 2011). Histopathological study showed an excellent consistency between the intracortical myelin mapping results and cortical cytoarchitectonic results (Glasser and Van Essen, 2011; Righart et al., 2017). Also, some recent work proposed the enhanced sensitivity of detecting changes in intracortical signal intensity associated well with the disease, such as multiple sclerosis, Parkinson disease, and normal subject with amyloid accumulation (Granberg et al., 2017; Righart et al., 2017; Yasuno et al., 2017). Thus, given that the T1W/T2W ratio is a clinically easy-to-implement measure, researchers regarded the technique as a parsimonious intracortical myelin marker (Du et al., 2019; Li et al., 2019).

On the other side, prior studies have been demonstrated that Fluorodeoxyglucose positron emission tomography (FDG PET) and gray matter (GM) atrophy are reproducible imaging biomarkers for AD continuum (Bloom, 2014; Kato et al., 2016). As the reliable synaptic dysfunction biomarker, FDG PET study demonstrated that AD dementia patients are characterized by hypometabolism in the parieotemporal association regions, posterior cingulate (PCC), and precuneus (PCu) (Lowe et al., 2018). Also, hypometabolism in the inferior parietal lobe (IPL), PCC/PCu and hippocampus (HP) may predict the likelihood of clinical progression from MCI to AD dementia, or from normal aging to MCI (Swaab, 1991; Jones et al., 2016; Lowe et al., 2018). As for gray matter atrophy, it has applied widely in AD continuum research. Converged evidence reveal the AD damage pattern, featured as temporal and parietal cortices atrophy or thinning. In particular, HP volume is the most validated biomarkers of AD progression (Jack et al., 2004). Emerging neuroimaging literature demonstrates the progressive HP atrophy from the AD preclinical individuals to AD dementia patients. Accordingly, to assess the sensitivity of the newly proposed T1W/T2W ratio for AD continuum depicting, considering FDG PET and gray matter atrophy results as reference are necessary.

The goal of our study was to detect the transformational patterns of the intracortical myelin content as AD progresses. The novelty and highlights of our investigation include that: (Braak et al., 2006) adopting the latest biological AD diagnosis criteria (Jack et al., 2018). To our best knowledge, except the work of our research team, few neuroimaging studies focusing the AD continuum under the biological research framework (Montal et al., 2017; Li et al., 2019; Kalaria, 2016) and we utilized the clinically accessible T1W/T2W ratio mapping method (Glasser and Van Essen, 2011; Ganzetti et al., 2015). Specifically, we identified 252 subjects in the AD continuum and used both the CSF and amyloid PET to determine the biological profile. To assess the sensitivity of this approach, we also analyzed the voxel-based morphometry and PET-based metabolism. Based on the myelin model of Bartzokis, regions with later myelination process would have less protection against damage factors (Bartzokis, 2004; Bartzokis et al., 2007; Phillips et al., 2016). Further, neuropathological evidence showed AD hallmark appear first in parietal and medial temporal and cortex (Braak et al., 2006). Accordingly, we hypothesized that the earliest demyelination processes might appear in the parietal neocortical regions in AD continuum.

## Materials and Methods

### Alzheimer’s Disease Neuroimaging Initiative

Data used in this study was obtained from the Alzheimer’s disease Neuroimaging Initiative (ADNI) database (adni.loni.usc.edu). The ADNI was launched in 2003 by the National Institute on Aging (NIA), the National Institute of Biomedical Imaging and Bioengineering (NIBIB), the Food and Drug Administration (FDA), private pharmaceutical companies and non-profit organizations, as a $60 million, 5-year public-private partnership. The primary goal of ADNI has been to test whether serial magnetic resonance imaging (MRI), positron emission tomography (PET), other biological markers, and clinical and neuropsychological assessment can be combined to measure the progression of mild cognitive impairment (MCI) and early Alzheimer’s disease (AD). Determination of sensitive and specific markers of very early AD progression is intended to aid researchers and clinicians in developing new treatments and monitor their effectiveness, as well as lessen the time and cost of clinical trials.

### Study Subjects Inclusion

All procedures conducted in our study involving human participants were following the ethical rules of the institutional and national research committee and with the 1975 Helsinki declaration and its later amendments or comparable ethical standards. The ADNI project was authorized by the Institutional Review Boards of all participating institution and informed written consent was obtained from participants at each site.

**FIGURE 1:**
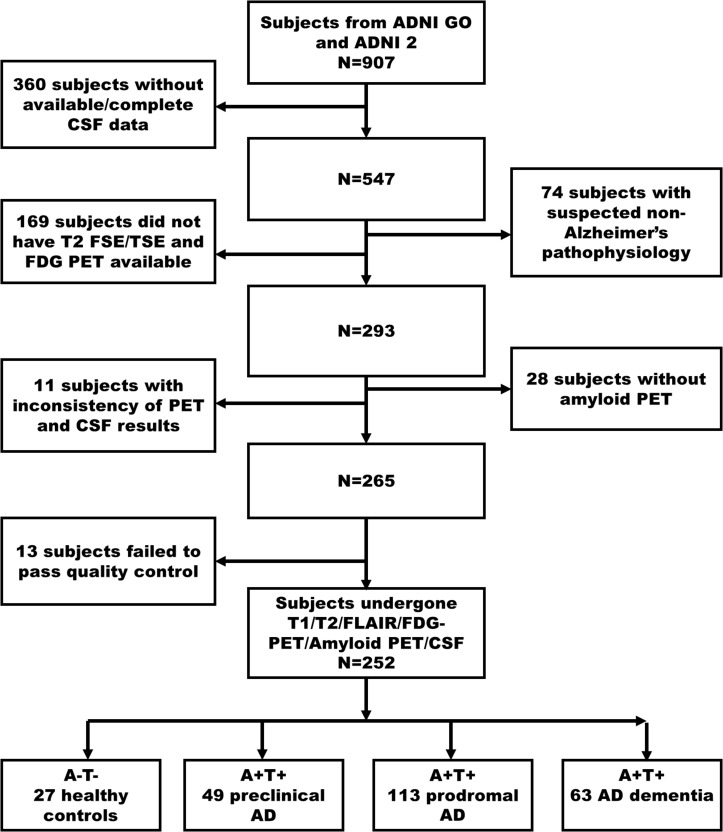
The flowchart illustrates inclusion procedures. Amyloid status positive (A+) defined as Aβ_1–42_<192 pg/ml combined with Florbetapir composite standardized uptake values >1.11; Tau status positive (T+) defined as p-Tau_181_ > 23 pg/ml. Firstly, we identified 907 subjects in ADNI 2/GO (2018 November). In a step-wise way, we excluded 360 subjects without available CSF data, 74 subjects with SNAP, 169 subjects without T2-weighted and FDG PET data, 28 subjects without amyloid PET, 11 subjects having inconsistency biological classification based on CSF (Aβ_1–42_) and amyloid PET, and 13 subjects having excessive head motion and severe image artifacts. Finally, we identified 27 cognitively unimpaired with normal AD biomarkers (A- T-, controls), 49 preclinical AD (A+ T+), 113 AD with MCI (A+ T+), and 63 AD dementia (A+ T+). AD, Alzheimer’s disease; MCI, mild cognitive impairment; FDG-PET, Fludeoxyglucose positron emission tomography; SNAP, suspected non-Alzheimer’s pathophysiology; ADNI, Alzheimer’s Disease Neuroimaging Initiative.

We searched the potential subjects from the ADNI GO/ADNI 2 database in 2018, November, and firstly found 907 subjects (Figure 1. Flowchart of inclusion). Subsequently, we excluded the subjects as following steps (Braak et al., 2006) without CSF data to determine the biological profile; (Kalaria, 2016) with the suspected non-Alzheimer’s pathophysiology (SNAP) (Dani et al., 2017; Jack et al., 2018) without available T1- and T2-weighted MRI, and FDG PET to conducted imaging analyses; (Perrotin et al., 2015) without amyloid PET to complete biological profile double-confirmation; (Peng et al., 2015) with inconsistent pathological profile (i.e., discrepant amyloid status of amyloid PET and CSF); (Stricker et al., 2016) fail to data quality controls (e.g., excessive head motion, suffering specific symptom or diseases). Finally, we identified 252 subjects who undergone simultaneous scan (i.e., the time interval of scan less than 4 weeks) of MRI and FDG PET, lumbar puncture, and comprehensive neuropsychological assessments.

### Subjects Classification Based on Cognitive Stage and Biological Profile

All participants underwent assessment of the general cognitive ability and disease severity (Mini-Mental State Examination, MMSE, and Clinical Dementia Rating, CDR, respectively), and other cognitive domains including memory (Wechsler Memory Scale LM and DM, immediate and delayed memory; Auditory Verbal Learning Test, AVLT, total and 30 min delayed score), processing speed (Trail-Making Test part A, TMT-A), visuospatial (Clock drawing Test, CDT), executive (TMT part B, TMT-B), and language ability (Category verbal fluency, CVF; Boston Naming Test, BNT).

Based on cognitive performance, we grouped subjects into cognitively unimpaired subjects, MCI, and dementia patients. Explicitly, we defined the cognitively unimpaired as subjects had CDR score of 0, MMSE between 24 and 30 (inclusive), DM ≥ 9 for subjects with ≥16 years of education; ≥5 for subjects with 8–15 years of education; and ≥3 for years of education ≤7; non-depression (Geriatric Depression Scale-15 score version <6) and non-dementia. We defined MCI as subjects preserved daily living activities, non-dementia, and objective cognitive impairment as shown on the DM and CDR score of 0.5. We defined dementia as: patients had MMSE score ≤ 26, CDR ≥ 0.5, and met the NINCDS/ADRDA criteria for probable AD.

Subsequently, we downloaded CSF dataset and identified the levels of Aβ1-42, total Tau (*T*-Tau) and phosphorylated tau (*p*-Tau181), which obtained using the standardized ADNI protocol as previously (Shaw et al., 2010). Based on previous ROC analyses of the autopsy-confirmed AD cases versus the NC group, we adopted the recommended cut-off of CSF biomarkers: 192 pg/ml for Aβ_1__–__42_ and 23 pg/ml for *p*-Tau_181_ (De Meyer et al., 2010; Shaw et al., 2010). Although the NIA-AA research framework suggests that either CSF or amyloid PET enables to determine one’s biological AD profile, in some cases, CSF Aβ_1__–__42_ might become abnormal before amyloid PET (Bateman et al., 2012; Ben Bouallegue et al., 2017). Based on these evidence, some previous studies suggested that the combination of amyloid CSF and PET biomarker may further improve the accuracy of biological classification to some extent (Bouter et al., 2019; Spallazzi et al., 2019). Thus, we adopted the recommended composite Florbetapir cutoff value of 1.11 (whole cerebellum as reference region) (i.e., A+ represents amyloid PET SUVR composite score > 1.11), which is equivalent to the upper 95% confidence interval above the mean of a group of young healthy controls (Landau et al., 2013). Finally, we excluded 11 subjects with the inconsistency results of amyloid status. We also excluded 74 subjects with normal Aβ_1__–__42_ and abnormal p-Tau_181_ (A−T+) since it might represent the non-AD related pathology.

Finally, we classified all subjects into four groups according to their cognitive performance and biological profile (Jack et al., 2018). Exactly, (a) controls: consisting of cognitively unimpaired subjects with normal Aβ_1__–__42_, *p*-Tau_181_ and negative amyloid status based on PET (A-T-); (b) preclinical AD, consisting of cognitively unimpaired subjects with abnormal Aβ_1__–__42_, normal p-Tau_181_, and positive amyloid status based on PET (A+T+); (c) prodromal AD, consisting of MCI with abnormal Aβ_1__–__42_, *p*-Tau_181_ and positive amyloid status based on PET (A+T+); (d) AD with dementia, consisting of demented subjects with abnormal Aβ_1__–__42_, P-Tau_181_ and positive amyloid status based on PET (A+T+).

### Genetic Data

Apolipoprotein (APOE) genotyping for each subject was performed as previously described (Saykin et al., 2010). In brief, genotyping of all subjects for APOE allele status was analyzed using DNA extracted from peripheral blood cells. The cells were collected in single EDTA plastic tubes (10 ml) and were sent via overnight delivery, at room temperature, to the University of Pennsylvania AD Biofluid Bank Laboratory.

### Image Acquisition

Details of the ADNI neuroimaging acquisition protocol are publicly available on the Laboratory of Neuroimaging (LONI) website. Structural images were acquired based on 3D Magnetization Prepared Rapid Acquisition Gradient Echo (MPRAGE) T1W sequence and fast spin-echo/turbo spin-echo (FSE/TSE) sequence. T1W image parameters: voxel size = 1.1 × 1.1 × 1.2 mm3; echo time (TE) = 2.98 ms; repetition time (TR) = 2300 ms. T2W image parameters: TR = 4000 ms; TE = 78 ms; voxel size = 0.9375 × 0.9375 × 4 mm3. The FLAIR images were obtained using an echo-planar imaging sequence with the following parameters: TR = 9000 ms, TE = 90 ms, and TI = 2500 ms. Meanwhile, FDG PET images were acquired by either a 30-min six frame scan acquired 30 to 60-min post-injection or a static 30-min single-frame scan acquired 30 to 60-min post-injection (Landau et al., 2011). Amyloid PET data were acquired during 4 × 5min time frames measured 50–70 min post-injection of ^18^F-AV45. More detailed information can be found online regarding PET scanning protocol: http://adni.loni.usc.edu/wp-content/uploads/2010/05/ADNI2_PET_Tech_Manual_0142011.pdf.

### Voxel-Based Morphometry Analysis

We processed the T1W images using the Computational Anatomy Toolbox (CAT, version 12^1^) in SPM12. Concisely, we firstly aligned the T1W images to the standardized template image. We segmented the standardized T1W images into the compartments of CSF, WM, and GM with the bias correction. Sequentially, we normalized the GM density maps to the MNI space as 1 × 1 × 1 mm^3^ voxels and smoothed using the 8 mm FWHM Gaussian kernel. Finally, we estimated the modulated GM maps.

### T1-Weighted/T2-Weighted Ratio Mapping

The T1W/T2W ratio mapping was processed on the basis of the workflow, MRtool^2^ implemented in the SPM12 software, described by Ganzetti et al. (2014). This standardized pipeline comprises the bias correction and intensity calibration on both the T1- and T2 weighted images and the subsequent computation of the ratio value between the pre-processed T1-weighted and T2-weighted images.

Specifically, the original T2W image was firstly co-registered to the T1W image through a rigid-body transformation. Next, we separately the subjected T1W and T2W images to bias correction. After correcting for the intensity non-uniformity, the T1W and T2W images were further processed to standardize their intensity by using a linear scaling procedure. After calibrating the T1W and T2W images, their ratio was calculated to generate the calibrated ratio map. To conduct two-sample *T*-test comparisons between groups, we spatially transformed the calibrated ratio map from the individual subject space to the Montreal Neurological Institute (MNI) space. According to the research hypothesis, we extracted the GM components of the T1w/T2w ratio maps based on the segmented normalized T1W image. Before the statistical analyses, we smoothed the T1W/T2W ratio map with a Gaussian kernel of 6 mm full-width at half maximum (FWMH).

### Fluorodeoxyglucose and Amyloid PET Analysis

We downloaded the ^18^F FDG PET and amyloid PET data from the ADNI database in its most processed format (PET Pre-processing protocol^3^, University of Berkeley). Specifically, these pre-processed scans were created following co-registration dynamic (to the first frame), averaging frames, spatial re-orientation (AC-PC line), intensity normalization (within subject-specific mask), and smoothing (uniform isotropic spatial resolution 8 mm FWMH). To exclude the between-subject nuisance variability in tracer uptake, we normalized the intensity of FDG PET by dividing it by the cerebellar pons/vermis; also, we normalized the intensity of amyloid PET by dividing it by the whole cerebellum. Next, the produced standardized uptake values (SUVR) maps entered the followed statistical analyses (Landau et al., 2011).

### White Matter Hyperintensities Segmentation and Quantification

Given that the white matter hyperintensities (WMH) may correlate with the demyelination and metabolism abnormalities, we compared the WMH load difference between groups in the current study (Jiaerken et al., 2018). For each subject, their 3D T1-weighted scans and FLAIR scans were normalized to MNI space. Then the Lesion Segmentation Toolbox (LST) in SPM12 was used to produce the WMH lesion segmentation maps based on the 3D T1-weighted scans and FLAIR scans (Schmidt et al., 2012). Automatically generated lesion maps were further manually reviewed for misclassification by two experienced neuroradiologists. Next, the WMH volume for the each subject was calculated by multiplying the voxel number by 1 mm^3^. More information can be obtained in our previous work (Luo et al., 2017, 2018).

### Statistics

We conducted the statistical analyses utilizing IBM SPSS 24 statistical software for Windows. Group differences in the age, education level, neuropsychological scores, and CSF were tested by the ANOVA, followed by *post hoc* two-sample *T*-tests if ANOVA was significant (*p* < 0.05, Bonferroni corrected).

We adopted the two-sample *T*-tests to examine the difference between controls and patient groups in the intracortical T1W/T2W ratio mapping, VBM, and FDG PET data. Notably, all imaging analyses were restrained within a standardized group gray matter (GM) mask, created from the CAT12 and SPM Masking Toolbox (Ridgway et al., 2009). In a voxel-wise way, we performed the imaging statistical in the DPABI, which is based on SPM 12 (Yan et al., 2016). To correct the multiple comparisons, we utilized the Gaussian random field (GRF) theory planted in the statistical module of the DPABI (Yan et al., 2016). Firstly, the p maps (statistical map) were converted to the Z maps according to the normal inverse cumulative distribution function, with the sign of group mean differences applied. The thresholding approach we used is single-voxel thresholds (cluster-defining thresholds) of *p* < 0.005 (*Z* > 2.58), and cluster size thresholds of *p* < 0.05 (Altman and Bland, 1994; Nichols and Hayasaka, 2003; Chen et al., 2018). Given the possible effects of age, gender, and APOE ε4 status, we also controlled these factors as covariates in the following analyses. Furthermore, given that the possible impacts of GM atrophy on T1W/T2W ratio, we also measured the group difference after controlling the GM segmented map in a voxel-wise way. We conducted these statistical analyses based on DPABI (Yan et al., 2016; Chen et al., 2018).

To investigate the possible pathological mechanism of imaging metrics, we performed partial correlation analyses among imaging metrics, neuropathological, and neuropsychological data. Specifically, we obtained the mean value of T1W/T2W ratio and FDG PET SUVR from regions showing significant between-group difference below. Based on SPSS, we performed a series of correlation analyses used age, gender, and APOE ε4 status as covariates and Bonferroni correction approach. The variables in correlation analyses included the mean value of T1W/T2W ratio, FDG PET SUVR, amyloid PET SUVR in bilateral inferior parietal lobe and hippocampus regions, CSF data (Aβ_1__–__42_, *T*-Tau, *p*-Tau_181_), general and multi-domain cognitive abilities score. After multiple comparison correction, some correlation relationship was no longer significant, we still showed these results in the manuscript and labeled them as “uncorrected” for the explorative purpose.

## Results

### Demographics and AD Biomarker Profile

We presented the quantitative variables as the mean and standard deviations (SD), and the categorical variable as absolute and relative frequency. There were no statistical differences in term of education level, gender composition, and white matter hyperintensities among four groups. Concerning the pair-wise comparison, preclinical AD only had inferior AVLT performance than controls. Table 1 summarizes the demographics, CSF biomarker levels, and neuropsychological evaluation of the subjects (Supplementary Materials 1–3 shows *post hoc* two-sample *T*-test).

**TABLE 1:** Demographics, neuropsychological scale, and cerebrospinal fluid data by diagnostic category.

	**Controls**	**Preclinical AD**	**Prodromal AD**	**AD dementia**	**F/χ^2^**	***p* value**
Number	27	49	113	63		
Age	73.5 ± 4.5	75.8 ± 5.9	72.8 ± 6.2	74.0 ± 6.6	2.9	<0.04
Gender (F/M)	16/11	30/19	49/64	27/36	6.4	0.4
APOE E4 (%)/N	7.4%/2	55.1%/27	74.3%/84	77.8%/49	49.8	<0.001
Education	16.8 ± 2.6	16.5 ± 2.5	16.4 ± 2.7	16.0 ± 6.6	0.7	0.6
**General cognition**						
MMSE	28.9 ± 1.6	29.0 ± 1.0	27.4 ± 2.3^∗∗^	23.3 ± 2.3^*⁣**^	94.4	<0.001
CDR	0	0 ± 0.1	0.5 ± 0.1^*⁣**^	0.8 ± 0.3^*⁣**^	273.5	<0.001
CDRSUM	0.1 ± 0.2	0.2 ± 0.6	1.7 ± 1.0^*⁣**^	4.3 ± 1.5^*⁣**^	196.7	<0.001
Memory						
IM	15.3 ± 2.8	13.7 ± 3.4	8.5 ± 3.9^*⁣**^	4.6 ± 2.6^*⁣**^	95.7	<0.001
DM	14.4 ± 2.8	12.5 ± 3.5	5.9 ± 4.3^*⁣**^	1.8 ± 2.1^*⁣**^	126.0	<0.001
AVLT	49.6 ± 9.5	42.1 ± 8.7^∗^	33.6 ± 10.5^*⁣**^	24.0 ± 7.0^*⁣**^	62.1	<0.001
AVLT 30 min	8.3 ± 3.9	6.6 ± 4.0	3.1 ± 3.8^*⁣**^	0.8 ± 1.4^*⁣**^	44.3	<0.001
**Attention**						
TMT-A	32.6 ± 8.5	36.2 ± 16.2	40.7 ± 18.7	61.6 ± 34.7^*⁣**^	17.5	<0.001
**Executive**						
TMT-B	79.7 ± 53.6	97.2 ± 67.1	117.5 ± 71.0	168.8 ± 103.5^*⁣**^	11.9	<0.001
**Language**						
CVT	20.2 ± 5.4	20.8 ± 5.7	17.2 ± 5.1^∗^	12.6 ± 5.1^*⁣**^	27.5	<0.001
BNT	28.4 ± 2.3	28.4 ± 1.8	26.4 ± 3.9	22.7 ± 5.4^*⁣**^	25.0	<0.001
**Visuospatial**						
CDT	4.67 ± 0.62	4.59 ± 0.10	4.27 ± 0.96	3.52 ± 1.49	12.91	<0.001
WMH	5.3 ± 8.2	10.4 ± 18.0	6.9 ± 7.6	8.2 ± 7.6	1.8	0.1
GDS	0.8 ± 0.8	0.8 ± 0.9	1.9 ± 1.5^*⁣**^	1.7 ± 1.4^∗^	11.8	<0.001
**CSF**						
Aβ_1__–__42_ (pg/ml)	228.0 ± 25.2	140.3 ± 23.1^*⁣**^	135.6 ± 23.2^*⁣**^	124 ± 19.0^*⁣**^	148.8	<0.001
*T*-Tau (pg/ml)	43.9 ± 10.4	86.1 ± 42.1^∗^	110.0 ± 66.4^*⁣**^	126.8 ± 66.2^*⁣**^	14.4	<0.001
*p*-Tau_181_ (pg/ml)	17.5 ± 3.9	54.7 ± 25.3^*⁣**^	58.0 ± 26.4^*⁣**^	64.0 ± 28.9^*⁣**^	22.6	<0.001

*APOE, apolipoprotein E; MMSE, mini-mental state examination; CDR, clinical dementia rating; IM, immediate memory; DM, delayed memory; AVLT, auditory verbal learning test; CVT, category verbal test; TMT-A/B, trail-making test, part A and B; BNT, Boston naming test; CDT, clock drawing test, WMH, white matter hyperintensities; GDS, geriatric depression scale; CSF, cerebral spinal fluid. ^∗^, ^∗∗^, ^∗∗∗^ represents *p* < 0.05, *p* < 0.01, *p* < 0.001, respectively, meaning patient group (preclinical AD, prodromal AD, and AD dementia) significantly different compared with controls.*

### T1W/T2W Ratio Value Decreases Start From Inferior Parietal Lobule in the AD Continuum

**FIGURE 2:**
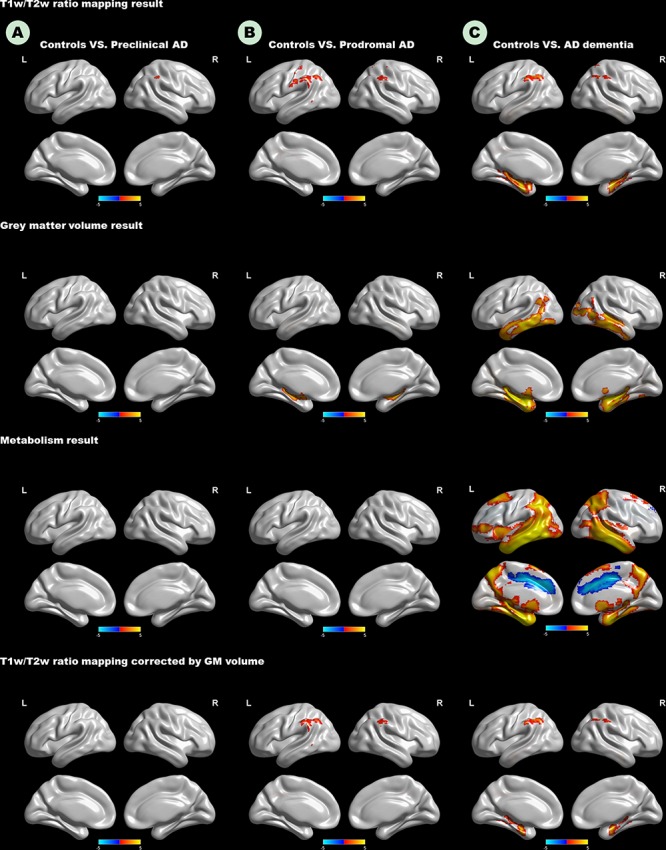
Top panel: T1W/T2W ratio change patterns in the AD continuum. Second row: Voxel-based morphometry change patterns in the AD continuum. Third row: FDG PET change patterns in the AD continuum; Bottom panel: T1W/T2W ratio change patterns in the AD continuum, corrected by gray matter volume in a voxel-wise way. **(A)** Difference in controls versus preclinical AD; **(B)** difference in controls versus prodromal AD; **(C)** difference in controls versus AD dementia. Only clusters that survive GRF corrected *p* < 0.005 (*Z* > 2.58) at height and *p* < 0.05 at cluster level are shown. Hot and cold color represents positive and negative significant values, respectively. T1W/T2W, T1-weighted/T2-weighted; IPL, inferior parietal lobule; AD, Alzheimer’s disease; FDG-PET, Fludeoxyglucose positron emission tomography; GRF, Gaussian random field.

**FIGURE 3:**
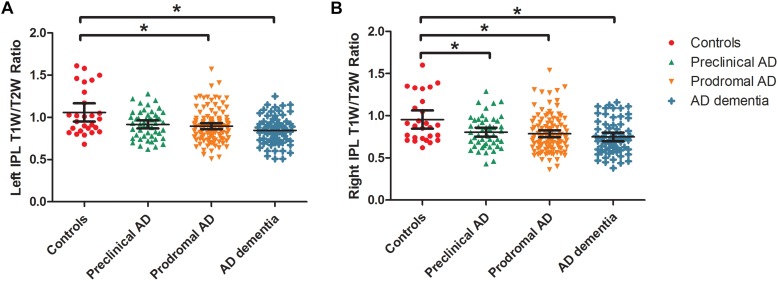
Box and whisker plots illustrate the T1W/T2W ratio change dynamics in left **(A)** and right **(B)** IPL in the AD continuum. We extracted the mean ratio value from the bilateral IPL based on the difference region between controls and AD dementia. The cognitively unimpaired preclinical AD subjects had a lower T1W/T2W ratio value in the right IPL than controls (corrected). As the disease progresses, prodromal AD subjects had a lower ratio value in the bilateral IPL regions than controls. T1W/T2W, T1-weighted/T2-weighted; IPL, inferior parietal lobule; AD, Alzheimer’s disease.

As expected, we observed that cognitively unimpaired preclinical AD subjects had lower T1W/T2W ratio value in the right inferior parietal lobule (IPL) than controls (Figure 2, top panel). As the disease progresses, prodromal AD subjects had a lower ratio value in two-sided IPL regions than controls. Ultimately, AD dementia had the most widespread T1W/T2W ratio decrease, involving the bilateral hippocampus (HP) and bilateral IPL, relative to controls (Figure 3). Controlling the GM volume in a voxel-wise way, no T1W/T2W ratio value differences existed between controls and cognitively unimpaired preclinical AD (Figure 2, bottom panel). But the prodromal AD subjects had decreased ratio value in the bilateral IP and left middle temporal gyrus compared to controls. Concerning AD dementia, the results remained almost the same. After taking the age, gender, and APOE ε4 status into consideration, significant difference regions become narrow, the ratio value difference between controls and preclinical AD no longer existed, and the right-side IPL differential region was no longer in existence. Supplementary Material 12 illustrates the imaging results corrected by age, gender and APOE ε4 status, and Supplementary Materials 4–7 shows the MNI coordinate information.

### Gray Matter Volume Atrophy Start From Hippocampus in the AD Continuum

The Figure 2 (second row) shows the GM volume group-difference maps. We found no gray matter volume differences between controls and preclinical AD. As the disease progresses, bilateral HP atrophy can be found in prodromal AD subjects, when compared to controls. At the stage of AD dementia, subjects had widespread GM atrophy relative to controls, particularly in the bilateral medial temporal lobe. After taking the age, gender, and APOE ε4 status into consideration, results remained almost unaltered (Supplementary Materials 8, 9).

### Widespread Metabolism Decrease in AD Dementia

Neither preclinical AD nor prodromal AD had decreased metabolism relative to controls (Figure 2). Only the AD dementia patients had increased metabolism in the left middle cingulate cortex, and widespread metabolism decrease compared to controls, especially in the bilateral middle temporal gyrus (MTG). After taking the age, gender, and APOE ε4 status into consideration, regions showing increased metabolism was no longer in existence (Supplementary Materials 10, 11).

### Correlation Analyses

**FIGURE 4:**
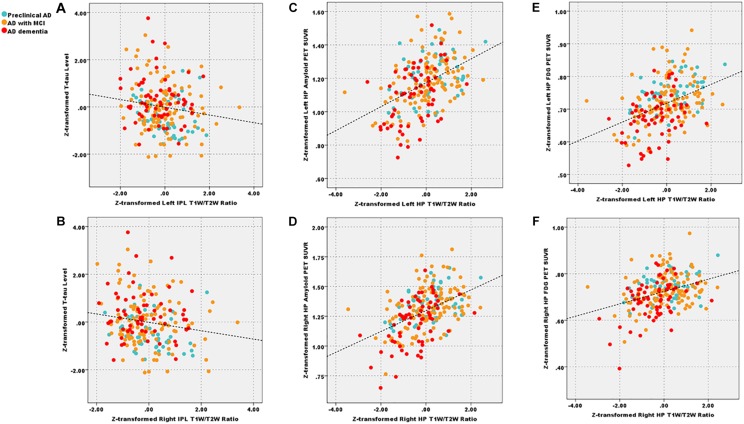
After corrected by the age, gender, and APOE ε4 status, decreased left and right IPL T1W/T2W ratio value (Z-transformed) related to total Tau level (**A**, *r* = −0.15; **B**, *r* = −0.17, *p* < 0.05 uncorrected) across patient groups; additionally, T1W/T2W ratio (Z-transformed) related to amyloid PET SUVR (Z-transformed) in left (**C**, *r* = 0.41, *p* < 0.05 Bonferroni corrected) and right HP (**D**, *r* = 0.43, *p* < 0.05 Bonferroni corrected); T1W/T2W ratio related to FDG-PET SUVR in left (**E**, *r* = 0.40, *p* < 0.05 Bonferroni corrected) and right (**F**, *r* = 0.38, *p* < 0.05 Bonferroni corrected) HP. IPL, inferior parietal lobule; HP, hippocampus; T1W/T2W, T1-weighted/T2-weighted; PET, Positron emission tomography; FDG-PET, Fludeoxyglucose PET; SUVR, standard uptake value ratio.

We showed the results centering on the T1W/T2W ratio and put completed correlation results in Supplementary Material 13. Within patient groups (A+T+), the left (*r* = −0.15) and right IPL (*r* = −0.17) T1W/T2W ratio value related to the *T*-tau level (*p* < 0.05 uncorrected), the left HP T1W/T2W ratio value related to the Aβ_1__–__42_ level (*r* = 0.14, *p* < 0.05, uncorrected). Within the patient groups, we also found that T1W/T2W ratio related to amyloid SUVR in the left (*r* = 0.41, *p* < 0.05 corrected) and right HP (*r* = 0.43, *p* < 0.05 corrected). However, no correlation relationship between the T1W/T2W ratio and the amyloid SUVR in IPL existed (Figure 4).

As for the clinical correlation relationships, within patient groups, we found that T1W/T2W ratio, FDG PET and HP volume correlated with general cognitive and multiple cognitive domains. These results suggested that all three metrics were effective imaging biomarkers. Further, all three metrics correlated with each other. Especially, the T1W/T2W ratio related to the FDG SUVR in term of left (*r* = 0.40, *p* < 0.05 corrected) and right (*r* = 0.38, *p* < 0.001) HP.

## Discussion

The purpose of our study was to detect the transformational patterns of the intracortical myelin content in patients in the AD continuum. First, we found the preclinical AD had the right IPL ratio value decrease, followed by bilateral IPL ratio decrease and bilateral HP volume atrophy in prodromal AD. As the disease progresses, AD dementia had the most widespread whole-brain atrophy, metabolism decrease, and decreased bilateral IPL and HP ratio value. Our study implied that the IPL demyelination process might represent one of the effective imaging biomarker indicating the onset of AD. Also, the T1W/T2W ratio mapping is a sensitive measure to assess the intracortical myelin content in AD continuum.

Relative to controls, we found that preclinical AD had the right IPL ratio value decrease relative to controls. As the disease progresses, the prodromal AD further had bilateral decreased IPL ratio. Formed by the angular gyrus and the supramarginal gyrus, the IPL highly connected with the prefrontal cortex, paralimbic cortex, and HP (Rushworth et al., 2006). Thus, the IPL involves many advanced cognitive functions, especially in term of spatial information processing, memory retrieval, and executive dysfunctions (Lindeboom and Weinstein, 2004; Cabeza, 2008). Also, the IPL is part of the default mode network (DMN); regions are active during rest but deactivate during external stimulation (Raichle et al., 2001). Due to the high processing load here, impairment of DMN would severely disturb the interconnectedness of intrinsic networks, thus is considered as the predictive biomarker for AD progression (Buckner et al., 2008). Based on the previously published agreement between T1W/T2W maps of myelin and the probabilistic cytoarchitectonics, we interpreted the decreased ratio value in IPL as myelin loss (Glasser and Van Essen, 2011). This hypothesis is alignment with the myelin model proposed by Bartzokis, suggesting that the late-myelinating regions, such as IPL, are vulnerable to a variety of insults (Bartzokis, 2004; Bartzokis et al., 2007).

Furthermore, we speculated AD-related pathologies accumulation and processing load shift constitute the damage to IPL (Jones et al., 2017). Supportive evidence from our results showed that decreased IPL ratio value related to increased total tau level within patients. Even after controlling age, gender, and APOE ε4 status, results remained mostly unchanged. Similarly, previous studies have reported that preclinical AD (amyloid positive healthy elderly) had increased Tau binding in IPL (Schultz et al., 2018). Thus, neurofibrillary tangles (NFT)-related toxicity may cause demyelination in these vulnerable regions (Bloom, 2014). However, without histological data or Tau PET imaging, such interpretations should be made with caution. Particularly, the correlation strength between decreased IPL ratio value and total Tau level is weak. On the other hand, another possible explanation is that cognitively intact preclinical AD may shift their processing load to the posterior DMN, regions serving as the core processing hub in the brain (Jones et al., 2017; Lowe et al., 2018), to avert system failure and clinical impairment from the degenerative process. Taken together, we inferred that direct AD-related pathologies and indirect “wear and tear effects” might jointly contribute to the IPL demyelination (Swaab, 1991).

Although the cross-sectional study design, we hypothesized that right IPL impairment might represent an earlier demyelination process than the left during disease progression. Given that the dominant laterality of visual-related process, involving the right IPL, thus we interpreted the extra processing load to contribute to the earlier myelin loss than its opposite side (Chance, 2014). Still, future longitudinal studies of T1W/T2W ratio mapping are needed to validate our hypothesis. After correcting the GM volume in a voxel-wise manner, the trend of decreased ratio value of IPL in prodromal AD remained. These results thus suggested that T1W/T2W ratio is a potential candidate marker of intracortical tissue integrity independent of atrophy.

Emerging evidence demonstrated that HP is intensively involved in AD progression (Aggleton et al., 2016; Mattsson et al., 2019). Consistently, we found prodromal AD patients (AD with MCI) had smaller bilateral HP volume than controls. Moreover, in alignment with the cascading network failure model, our results supported that posterior DMN undertook extra processing load from the stressed system vulnerable to Tau-related pathologies (Jones et al., 2016). Thus IPL demyelination may appear earlier than HP atrophy in preclinical AD subjects (Jones et al., 2017). However, given that HP is not a homogenous structure, thus subfield volume with higher sensitivity should be investigated in the future (Small et al., 2011). Subsequent correlation analysis showed HP volume related with both the amyloid deposition and total Tau within patients. These correlations are supported by previous pathological evidence that the accumulation of Tau protein highly linked with each other in the temporal lobe (Marks et al., 2017). Based on previous evidence that Tau in the medial temporal lobe strongly associated with GM volume atrophy, we thus inferred the HP atrophy in prodromal AD may attributed to NFT-related toxicity (Sepulcre et al., 2016).

As the disease progresses to the final stage, we noticed AD dementia had limited T1W/T2W ratio decrease, only involving bilateral IPL and HP. However, according to previous work, AD dementia patients had widespread demyelination (Bartzokis, 2004; Phillips et al., 2016). Thus, this result was partly opposed to our expectations. The possible reason is that AD dementia patients in our study had relatively mild disease severity (mean MMSE = 23.3 ± 2.3; mean global CDR = 0.8 ± 0.3). Consequently, there may still have time from the full spreading of pathologies. The interpretation supported by our VBM results that AD patients also had limited atrophy, merely involved in the bilateral temporal lobe. Also, the low specificity of T1W/T2W ratio may contribute to this result. To be specific, the amyloid-related iron deposition in AD dementia patients may cause the T1W/T2W ratio increase and then neutralize the demyelination-related T1W/T2W ratio decrease (Righart et al., 2017; Yasuno et al., 2017). In addition, previous studies have demonstrated that the regions that amyloid plaques localized with iron deposition are consistent with the AD pathological distribution pattern (Raha et al., 2013). The subsequent correlation analysis supports our interpretations to some extent, in detail, the T1W/T2W ratio positively related to the amyloid PET SUVR in bilateral HP. Notably, the unexpected correlation results are similar to previous results (Yasuno et al., 2017). However, it still should be noted that no correlation relationship between the IPL T1W/T2W ratio and amyloid PET SUVR was found. Given that the non-uniform distribution of myelin in the human brain, we inferred that the T1W/T2W ratio decrease in different regions might represent different pathological processes. Conclusively, future T1W/T2W studies combined with iron mapping technique and amyloid PET are urgent to validate these speculations.

Concerning FDG PET results, our results showed that only in AD dementia, rather than preclinical and prodromal AD, had widespread hypometabolism than the control group. FDG PET thus might have higher sensitivity in assessing disease severity in the later stage than T1W/T2W ratio mapping, because restricted regions showed decreased ratio value in AD dementia patients. These results demonstrated that these two methods might have complementary advantages in depicting AD progression. The following correlation analyses showed that both the T1W/T2W ratio and FDG PET linked with global and multi-domain cognitive ability across groups, suggested that two metrics are effective imaging biomarkers reflecting clinical symptom severity of AD. Additionally, T1W/T2W ratio strongly related to FDG PET in bilateral HP regions. We thus assumed that complex pathological process, such as tracts demyelination and neuronal synergic loss, might collectively contribute to cognitive impairment in patients at the later stage of AD continuum.

There are several strengths of our study, including the application of strict biological AD diagnosis criteria, consideration of the potential effects of cerebral small vascular disease (WMH load matched between groups), and the inclusion of multiple levels of biological metrics, particularly the neuropathological data. Also, there existed several limitations to this study. First, due to an insufficient sample, no subjects with A + T- biomarker profile included in this study. However, previous similar AD continuum studies have reported that there are biphasic trajectory changes in cortical thickness from A-T- to A + T- until A + T + stage in cognitively intact elderly (Montal et al., 2017). Therefore, the potential biphasic trajectory changes in ratio maps may be left out here. Second, our T2W images had coarser spatial resolution than T1W images. Therefore the possible partial volume effect (PVE) may affect the GM signal intensity (Ganzetti et al., 2014). Future ratio mapping studies with the same isotropic spatial resolution of the T1W and T2W images is thus required. Third, although the cortical T1W/T2W ratio is a parsimonious marker for neurodegenerative disease, however, it is still clear that how accurately it represents myelin contents (Righart et al., 2017; Yasuno et al., 2017; Du et al., 2019). Thus, it is possible that some pathological changes (iron deposition, free water or fiber density), other than demyelination, may also change T1W/T2W ratio. Future studies should combine this technique with other imaging modalities, such as neurite orientation and dispersion density imaging (NODDI) and quantitative susceptibility mapping (QSM) (Liu et al., 2015; Tariq et al., 2016), to improve the pathological specificity.

## Conclusion

Our study implied that the demyelination of the right IPL might represent the early AD-related abnormality in the disease continuum, showing more sensitivity than both the gray matter atrophy and hypometabolism. Additionally, we proved that T1W/T2W ratio mapping is an easy-to-implement and sensitive measure to assess the intracortical myelin content in the AD continuum.

## Data Availability

Publicly available datasets were analyzed in this study. This data can be found at: http://adni.loni.usc.edu.

## Author Contributions

XL, KL, QZ, YJ, and PH conceived and designed the study, and analyzed and interpreted the data. SW, ZS, XX, MZ, JX, CW, LK, and JZ critically revised the manuscript for intellectual content and performed the statistical analysis.

## Conflict of Interest Statement

The authors declare that the research was conducted in the absence of any commercial or financial relationships that could be construed as a potential conflict of interest.

## Footnotes

1 http://dbm.neuro.uni-jena.de/cat/index.html

2 https://www.nitrc.org/projects/mrtool/

3 http://adni.loni.usc.edu/methods/pet-analysis/pre-processing/
