# Supplementary material for: Application of T1-/T2-Weighted Ratio Mapping to Elucidate Intracortical Demyelination Process in the Alzheimer’s Disease Continuum
Source: Front Neurosci. 2019 Sep 10;13:904. doi: 10.3389/fnins.2019.00904 (PMC6748350; doi:10.3389/fnins.2019.00904)
Supplement: Supplementary file 1 [file Data_Sheet_1.PDF]

Application of T1- /T2-weighted ratio mapping to elucidate intracortical demyelination process along the continuum of the Alzheimer's disease defined by the biological classification

Author: Xiao Luo[1#], Kaicheng Li[1#], Qingze Zeng[1], Peiyu Huang[1], Yeerfan Jiaerken[1], Shuyue Wang[1], Ruiting Zhang[1], Tiantian Qiu[1], Zhujing Shen[1], Xiaojun Xu[1], Jingjing Xu[1], Chao Wang[1], Jiong Zhou[2*], Minming Zhang[1*], for the Alzheimer's Disease Neuroimaging Initiative (ADNI)

[1]Department of Radiology, The 2nd Affiliated Hospital of Zhejiang University School of Medicine, Hangzhou, China

[2]Department of Neurology, The 2nd Affiliated Hospital of Zhejiang University School of Medicine, Hangzhou, China

[#]Joint First authors, contribute equally to this study

***Joint senior authors**

Correspondence to:

Prof. Minming Zhang, MD, Ph.D.; Department of Radiology, The 2nd Affiliated Hospital of Zhejiang University, School of Medicine, No.88 Jie-Fang Road, Shang-Cheng District, Hangzhou, China, 310009; Phone: 86-0571-87315255; Fax: 86-0571-87315255; Email address: zhangminming@zju.edu.cn

Co-investigator:

The data used in the preparation of this article were obtained from the Alzheimer's Disease Neuroimaging Initiative (ADNI) database (www.adni.loni.usc.edu). As such, the investigators within the ADNI contributed to the design and implementation of ADNI and provided data but did not participate in the analysis or writing of this report. A complete listing of the ADNI investigators can be found in the supplement.

Supplementary Material 1. Comparison of demographics, neuropsychological scale, and cerebrospinal fluid data between controls and cognitively unimpaired preclinical AD

	Controls	Preclinical AD	T/χ^2	p value
**Number**	27	49		
**Age**	73.5±4.5	75.8±5.9	-1.8	0.1
**Gender (F/M)**	16/11	30/19	0	1.00
**APOE ε4 (%)/N**	7.4% /2	55.1%/27	16.8	<0.001
**Education**	16.8±2.6	16.5±2.5	0.6	0.6
**General cognition**				
**MMSE**	28.9±1.6	29.0±1.0	-0.1	0.9
**CDR**	0	0±0.1	-1.5	0.1
**CDRSUM**	0.1±0.2	0.2±0.6	-1.0	0.4
**Memory**				
**IM**	15.3±2.8	13.7±3.4	2.1	<0.05
**DM**	14.4±2.8	12.5±3.5	2.4	<0.05
**AVLT**	49.6±9.5	42.1±8.7	3.5	<0.001
**AVLT 30min**	8.3±3.9	6.6±4.0	1.7	0.1
**Attention**				
**TMT-A**	32.6±8.5	36.2±16.2	-1.1	0.3
**Executive**				
**TMT-B**	79.7±53.6	97.2±67.1	-1.2	0.3
**Language**				
**CVT**	20.2±5.4	20.8±5.7	-0.5	0.7
**BNT**	28.4±2.3	28.4±1.8	0.2	0.8
**Visuospatial**				
**CDT**	4.67±0.6	4.59±0.10	0.5	0.7
**WMH**	5.3±8.2	10.4±18.0	-1.4	0.2
**GDS**	0.8±0.8	0.8±0.9	0.1	0.9
**CSF**				
**Aβ_{1-42} (pg/ml)**	228.0±25.2	140.3±23.1	15.3	<0.001
**T-Tau (pg/ml)**	43.9±10.4	86.1±42.1	-7.6	<0.001
**p-Tau$_{181}$(pg/ml)**	17.5±3.9	54.7±25.3	-5.1	<0.001

Abbreviations: APOE, Apolipoprotein E; MMSE, Mini-Mental State Examination; CDR, clinical dementia rating; IM, immediate memory; DM, delayed memory; AVLT, auditory verbal learning test; CVT, category verbal test; TMT-A/B, trail-making test, part A and B; BNT, Boston naming test; CDT, clock drawing test, WMH, white matter hyperintensities; GDS, geriatric depression scale; CSF, cerebral spinal fluid.

Supplementary Material 2. Comparison of demographics, neuropsychological scale, and cerebrospinal fluid data between controls and prodromal AD

	**Controls**	**Prodromal AD**	**T/χ^2**	**p value**
**Number**	27	113		
**Age**	73.5±4.5	72.8±6.2	0.6	0.6
**Gender (F/M)**	16/11	49/64	2.2	0.2
**APOE ε4 (%)/N**	7.4% /2	74.3%/84	41.2	<0.001
**Education**	16.8±2.6	16.4±2.7	0.8	0.4
**General cognition**				
**MMSE**	28.9±1.6	27.4±2.3	3.4	<0.001
**CDR**	0	0.5±0.1	-38.4	<0.001
**CDRSUM**	0.1±0.2	1.7±1.0	-8.5	<0.001
**Memory**				
**IM**	15.3±2.8	8.5±3.9	8.5	<0.001
**DM**	14.4±2.8	5.9±4.3	9.7	<0.001
**AVLT**	49.6±9.5	33.6±10.5	7.2	<0.001
**AVLT 30min**	8.3±3.9	3.1±3.8	6.2	<0.001
**Attention**				
**TMT-A**	32.6±8.5	40.7±18.7	-2.2	<0.05
**Executive**				
**TMT-B**	79.7±53.6	117.5±71.0	-2.6	<0.05
**Language**				
**CVT**	20.2±5.4	17.2±5.1	2.8	<0.01
**BNT**	28.4±2.3	26.4±3.9	2.7	<0.01
**Visuospatial**				
**CDT**	4.67±0.6	4.27±1.0	2.0	<0.05
**WMH**	5.3±8.2	6.9±7.6	-1.0	0.3
**GDS**	0.8±0.8	1.9±1.5	-3.7	<0.001
**CSF**				
**$A\beta_{1-42}$ (pg/ml)**	228.0±25.2	135.6±23.2	18.3	<0.001
**T-Tau (pg/ml)**	43.9±10.4	110.0±66.4	-7.9	<0.001
**$p\text{-}Tau_{181}$ (pg/ml)**	17.5±3.9	58.0±26.4	-5.1	<0.001

Abbreviations: APOE, Apolipoprotein E; MMSE, Mini-Mental State Examination; CDR, clinical dementia rating; IM, immediate memory; DM, delayed memory; AVLT, auditory verbal learning test; CVT, category verbal test; TMT-A/B, trail-making test, part A and B; BNT, Boston naming test; CDT, clock drawing test, WMH, white matter hyperintensities; GDS, geriatric depression scale; CSF, cerebral spinal fluid.

Supplementary Material 3. Comparison of demographics, neuropsychological scale, and cerebrospinal fluid data between controls and AD dementia

	Controls	AD with dementia	F/χ^2	p value
**Number**	27	63		
**Age**	73.5±4.5	74.0±6.6	-0.4	0.7
**Gender (F/M)**	16/11	27/36	2.0	0.2
**APOE ε4 (%)/N**	7.4% /2	77.8%/49	38.1	<0.001
**Education**	16.8±2.6	16.0±6.6	1.4	0.2
**General cognition**				
**MMSE**	28.9±1.6	23.3±2.3	11.6	<0.001
**CDR**	0	0.8±0.3	-14.5	<0.001
**CDRSUM**	0.1±0.2	4.3±1.5	-14.9	<0.001
**Memory**				
**IM**	15.3±2.8	4.6±2.6	17.5	<0.001
**DM**	14.4±2.8	1.8±2.1	23.4	<0.001
**AVLT**	49.6±9.5	24.0±7.0	14.2	<0.001
**AVLT 30min**	8.3±3.9	0.8±1.4	13.5	<0.001
**Attention**				
**TMT-A**	32.6±8.5	61.6±34.7	-4.3	<0.001
**Executive**				
**TMT-B**	79.7±53.6	168.8±103.5	-4.2	<0.001
**Language**				
**CVT**	20.2±5.4	12.6±5.1	6.5	<0.001
**BNT**	28.4±2.3	22.7±5.4	5.4	<0.001
**Visuospatial**				
**CDT**	4.67±0.6	3.52±1.5	3.8	<0.001
**WMH**	5.3±8.2	8.2±7.6	-1.7	0.1
**GDS**	0.8±0.8	1.7±1.4	-3.3	<0.005
**CSF**				
**$A\beta_{1-42}$ (pg/ml)**	228.0±25.2	124±19.0	21.5	<0.001
**T-Tau (pg/ml)**	43.9±10.4	126.8±66.2	-8.3	<0.001
**$p\text{-}Tau_{181}$ (pg/ml)**	17.5±3.9	64.0±28.9	-6.5	<0.001

Abbreviations: APOE, Apolipoprotein E; MMSE, Mini-Mental State Examination; CDR, clinical dementia rating; IM, immediate memory; DM, delayed memory; AVLT, auditory verbal learning test; CVT, category verbal test; TMT-A/B, trail-making test, part A and B; BNT, Boston naming test; CDT, clock drawing test, WMH, white matter hyperintensities; GDS, geriatric depression scale; CSF, cerebral spinal fluid.

Supplementary Material 4. T1-Weighted/T2-Weighted ratio value decrease in the AD continuum

Region	MNI coordinate			Peak intensity
	X	Y	Z	
Controls VS. Preclinical AD				
Right Inferior Parietal Lobule	57	-42	45	3.9
Controls VS. Prodromal AD				
Left Middle Temporal Gyrus	-69	-33	-6	4.2
Left Inferior Parietal Lobule	-54	-42	51	4.1
Right Inferior Parietal Lobule	63	-36	39	4.1
Controls VS. AD dementia				
Left Hippocampus	-27	-39	0	5.5
Right Hippocampus	33	-36	-6	5.7
Left Inferior Parietal Lobule	-54	-45	48	5.1
Right Inferior Parietal Lobule	54	-39	51	4.1

The thresholding approach we used is single-voxel thresholds (cluster-defining thresholds) of $p<0.005$ ($Z>2.58$), and cluster size thresholds of $p<0.05$. Abbreviations: AD, Alzheimer's disease; Controls: cognitively unimpaired with normal AD biomarker profile.

Supplementary Material 5. T1-Weighted/T2-Weighted ratio value decrease in the AD continuum, results corrected by grey matter in a voxel-wise way

**Region**	**MNI coordinate**			**Peak intensity**
	**X**	**Y**	**Z**	
Controls VS. Preclinical AD				
None	/	/	/	/
Controls VS. Prodromal AD				
Left Middle Temporal Gyrus	-66	-36	-3	4.1
Left Inferior Parietal Lobule	-54	-42	51	4
Right Inferior Parietal Lobule	63	-36	39	4.1
Controls VS. AD dementia				
Left Hippocampus	-27	-39	0	5.6
Right Hippocampus	33	-36	-6	5.6
Left Inferior Parietal Lobule	-54	-45	48	5.1
Right Inferior Parietal Lobule	48	-51	51	3.9

The thresholding approach we used is single-voxel thresholds (cluster-defining thresholds) of $p<0.005$ ($Z>2.58$), and cluster size thresholds of $p<0.05$. Abbreviations: AD, Alzheimer's disease; Controls: cognitively unimpaired with normal AD biomarker profile.

Supplementary Material 6. T1-Weighted/T2-Weighted ratio value decrease in the AD continuum, corrected by age, gender and APOE ε4 status

Region	MNI coordinate			Peak intensity
	X	Y	Z	
Controls VS. Preclinical AD				
None	/	/	/	/
Controls VS. Prodromal AD				
Left Superior Mariginal Gyrus	-60	-21	36	4.0
Left Postcentral Gyrus	-54	-12	48	3.9
Controls VS. AD dementia				
Left Hippocampus	-30	-36	-3	5.1
Right Hippocampus	33	-36	-6	5.7
Right Inferior Parietal Lobule	54	-39	51	4.2

The thresholding approach we used is single-voxel thresholds (cluster-defining thresholds) of $p<0.005$ ($Z>2.58$), and cluster size thresholds of $p<0.05$. Abbreviations: AD, Alzheimer's disease; Controls: cognitively unimpaired with normal AD biomarker profile.

Supplementary Material 7. T1-Weighted/T2-Weighted ratio value decrease in the AD continuum, corrected by age, gender, APOE ε4 status, and grey matter in a voxel-wise way

Region	MNI coordinate			Peak intensity
	X	Y	Z	
Controls VS. Preclinical AD				
None	/	/	/	/
Controls VS. Prodromal AD				
Left Middle Temporal Gyrus	-66	-27	-12	3.4
Controls VS. AD dementia				
Left Hippocampus	-27	-39	0	5.2
Right Hippocampus	33	-36	-3	5.5
Left Caudate	-9	12	9	5.1
Right Superior Temporal Gryus	54	-21	12	4.5
Right Superior Mariginal Gyrus	45	-42	42	4.2

The thresholding approach we used is single-voxel thresholds (cluster-defining thresholds) of $p<0.005$ ($Z>2.58$), and cluster size thresholds of $p<0.05$. Abbreviations: AD, Alzheimer’s disease; Controls: cognitively unimpaired with normal AD biomarker profile.

Supplementary Material 8. Grey matter volume decrease in the AD continuum

Region	MNI coordinate			Peak intensity
	X	Y	Z	
Controls VS. Preclinical AD				
None	/	/	/	
Controls VS. Prodromal AD				
Left Hippocampus	-16.5	-6	-9	4.8
Right Hippocampus	31.5	-37.5	3	4.4
Controls VS. AD dementia				
Left Temporal Lobe*	-16.5	-7.5	-10.5	9.9

The thresholding approach we used is single-voxel thresholds (cluster-defining thresholds) of $p<0.005$ ($Z>2.58$), and cluster size thresholds of $p<0.05$. Abbreviations: AD, Alzheimer's disease; Controls: cognitively unimpaired with normal AD biomarker profile. *clusters involve bilateral inferior temporal gyrus, bilateral middle temporal gyrus, bilateral fusiform gyrus, and bilateral hippocampus.

Supplementary Material 9. Grey matter volume decrease in the AD continuum, corrected by age, gender and APOE ε4 status

**Region**	**MNI coordinate**			**Peak intensity**
	**X**	**Y**	**Z**	
Controls VS. Preclinical AD				
None	/	/	/	
Controls VS. Prodromal AD				
Left Hippocampus	-16.5	-4.5	-10.5	4.8
Right Hippocampus	31.5	-9	-15	4.1
Controls VS. AD dementia				
Left Temporal Lobe*	-16.5	-7.5	-10.5	8.5

The thresholding approach we used is single-voxel thresholds (cluster-defining thresholds) of $p<0.005$ ($Z>2.58$), and cluster size thresholds of $p<0.05$. Abbreviations: AD, Alzheimer's disease; Controls: cognitively unimpaired with normal AD biomarker profile. *clusters involve bilateral inferior temporal gyrus, bilateral middle temporal gyrus, bilateral fusiform gyrus, and bilateral hippocampus.

Supplementary Material 10. FDG SUVR changes in the AD continuum

Region	MNI coordinate			Peak intensity
	X	Y	Z	
Controls VS. Preclinical AD				
None	/	/	/	
Controls VS. Prodromal AD				
None	/	/	/	
Controls VS. AD dementia				
Left Inferior Temporal Gyrus	-54	-48	-18	7.6
Left Middle Cinguate Gyrus	-9	21	33	-4.9
Left Middle Frontal Gyrus	-21	21	60	6.1

The thresholding approach we used is single-voxel thresholds (cluster-defining thresholds) of $p<0.005$ ($Z>2.58$), and cluster size thresholds of $p<0.05$. Abbreviations: SUVR, standardized uptake values ratios; AD, Alzheimer's disease; Controls: cognitively unimpaired with normal AD biomarker profile.

Supplementary Material 11. FDG SUVR changes in the AD continuum, corrected by age, gender and APOE ε4 status

**Region**	**MNI coordinate**			**Peak intensity**
	**X**	**Y**	**Z**	
Controls VS. Preclinical AD				
None	/	/	/	
Controls VS. Prodromal AD				
None	/	/	/	
Controls VS. AD dementia				
Left Inferior Temporal Gyrus	-54	-48	-15	6.9

The thresholding approach we used is single-voxel thresholds (cluster-defining thresholds) of $p<0.005$ ($Z>2.58$), and cluster size thresholds of $p<0.05$. Abbreviations: SUVR, standardized uptake values ratios; AD, Alzheimer's disease; Controls: cognitively unimpaired with normal AD biomarker profile.

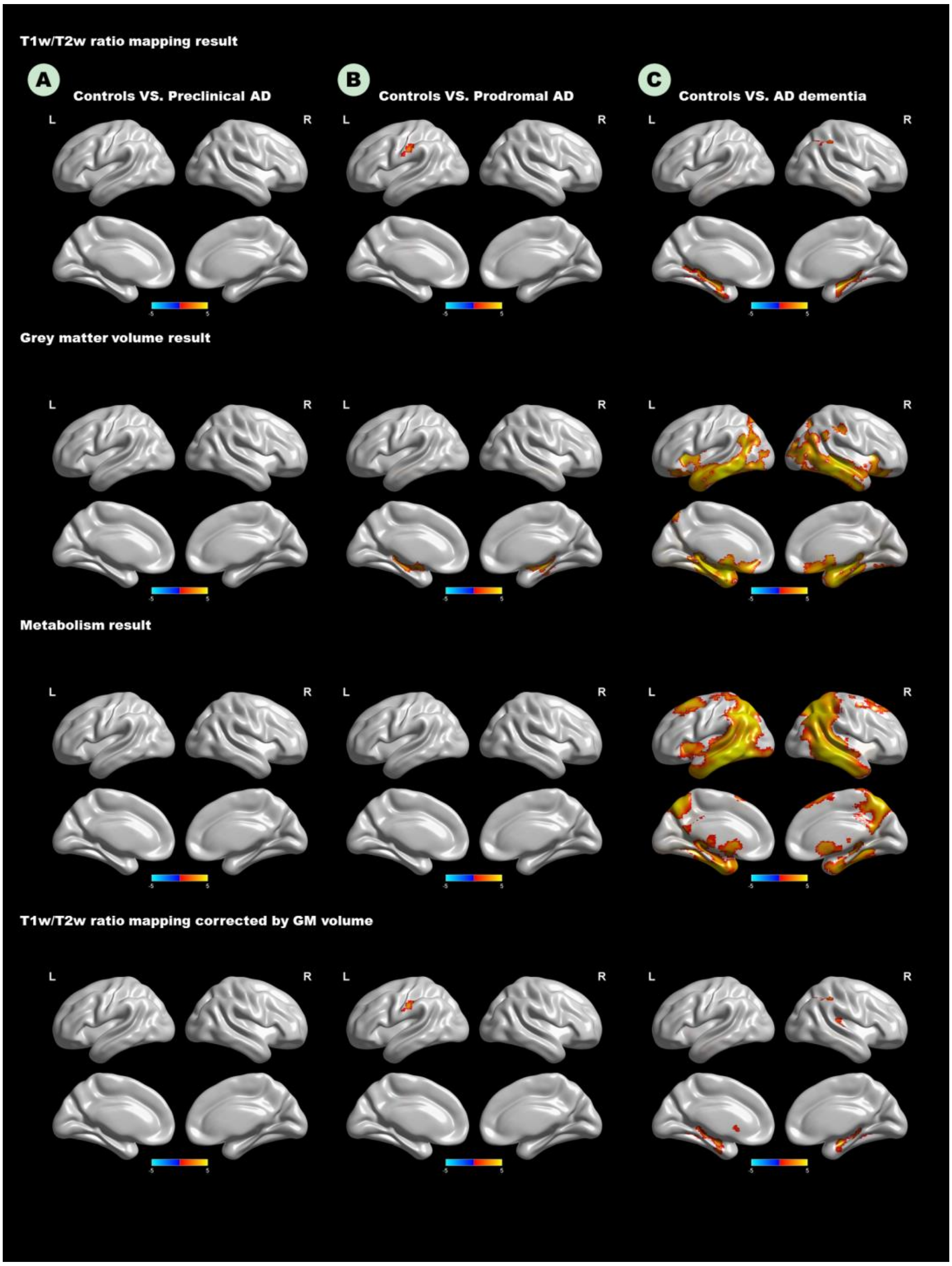

Supplementary Material 12

Top panel: T1-weighted/T2-weighted (T1W/T2W) ratio change patterns in the Alzheimer's disease (AD) continuum, corrected by age, gender, APOE ε4 status. Second row: Voxel-based morphometry change patterns in the AD continuum. Third row: FDG PET change patterns in the AD continuum; Bottom panel: T1-weighted/T2-weighted (T1W/T2W) ratio change patterns in the Alzheimer's disease (AD) continuum, corrected by grey matter volume in a voxel-wise way. (A) Difference in controls versus preclinical AD; (B) difference in

controls versus prodromal AD; (C) difference in controls versus AD dementia. The thresholding approach we used is single-voxel thresholds (cluster-defining thresholds) of $p<0.005$ ($Z>2.58$), and cluster size thresholds of $p<0.05$. Hot and cold color represents the positive and negative significant values, respectively.

Supplementary Material 13

To investigate the possible pathological mechanism of imaging metrics, we obtained mean values from regions with the difference between groups and related these values with neuropathological and neuropsychological data. In correlation analyses, we used age, gender, and APOE ε4 status as covariates and Bonferroni correction approach.

Details of correlation relationship between imaging metrics and neuropsychological data were shown below. Across patient groups, our correlation analyses results ($p<0.05$, Bonferroni corrected) showed that left HP T1W/T2W ratio value related to general cognitive (MMSE, r=0.34), visuospatial (CDT, r=0.27), memory (IM, r=0.37; DM, r=0.36; AVLT, r=0.35), language (CVT, r=0.32; BNT, r=0.44), executive function (TMT-B, r=-0.23). The right HP ratio value related to general cognitive (MMSE, r=0.29), visuospatial (CDT, r=0.26), memory (IM, r=0.35; DM, r=0.33; AVLT, r=0.30), language (CVT, r=0.29; BNT, r=0.37), executive function (TMT-B, r=-0.22). The left IPL T1W/T2W ratio value related to visuospatial (CDT, r=0.27), memory (IM, r=0.37; DM, r=0.36; AVLT, r=0.35), language (CVT, r=0.32, $p<0.001$; BNT, r=0.44, $p<0.001$), executive function (TMT-B, r=-0.23, $p<0.001$). All the results corrected by age, gender and APOE ε4 status.

Across patient groups, our correlation analyses results ($p<0.05$, Bonferroni corrected) showed that left HP FDG SUVR related to general cognitive (MMSE, r=0.41), visuospatial (CDT, r=0.24,), memory (IM, r=0.37; DM, r=0.37; AVLT, r=0.32), language (CVT, r=0.25; BNT, r=0.41). The right HP FDG SUVR related to general cognitive (MMSE, r=0.27), memory (IM, r=0.26; DM, r=0.27; AVLT, r=0.21), language (BNT, r=0.27), executive function (TMT-B, r=-0.22). The left IPL FDG SUVR related to general cognitive (MMSE, r=0.35), visuospatial (CDT, r=0.36), memory (IM, r=0.29; DM, r=0.30; AVLT, r=0.27), language (CVT, r=0.39; BNT, r=0.35), attention (TMT-A, r=-0.32). The right IPL FDG SUVR related to general cognitive (MMSE, r=0.26), visuospatial (CDT, r=0.25), memory (IM, r=0.22; DM, r=0.27; AVLT, r=0.23), language (CVT, r=0.30; BNT, r=0.26), attention (TMT-A, r=-0.30).
